# Graphene Oxide Demonstrates Experimental Confirmation of Abraham Pressure on Solid Surface

**DOI:** 10.1038/srep42538

**Published:** 2017-02-13

**Authors:** Anirban Kundu, Renu Rani, Kiran S. Hazra

**Affiliations:** 1 Institute of Nano Science and Technology, Habitat Centre, Phase 10, Sector 64, Mohali, Punjab, 160062, India

## Abstract

The century-old controversy over two contradicting theories on radiation pressure of light proposed by Abraham and Minkowski can come to an end if there is a direct method to measure the surface deformation of the target material due to momentum transfer of photons. Here we have investigated the effect of radiation pressure on the surface morphology of Graphene Oxide (GO) film, experienced due to low power focused laser irradiation. In-depth investigation has been carried out to probe the bending of the GO surface due to radiation pressure by Atomic Force Microscopy (AFM) and subsequently the uniaxial strain induced on the GO film has been probed by Raman Spectroscopy. Our results show GO film experience an inward pressure due to laser radiation resulting in inward bending of the surface, which is consistent with the Abraham theory. The bending diameter and depth of the irradiated spot show linear dependence with the laser power while an abrupt change in depth and diameter of the irradiated spot is observed at the breaking point. Such abrupt change in depth is attributed to the thinning of the GO film by laser irradiation.

Experimental evidence in support of the existence of radiation pressure of light is one of the well-known controversial topic in physics^1^. Contradicting theories established by Minkowski^2^ and Abraham^3^ about the momentum of light have initiated this debate. Minkowski’s electromagnetic theory explains that within a dielectric medium of refractive index *n*, the energy of light pulse can be defined by *nE/c*, whereas according to Abraham’s theory it is equal to *E/nc*. Due to the difference in consideration of light momentum in Abraham and Minkowski model, the direction of interface movement by radiation pressure on the dielectric medium becomes opposite to each other. According to Ashkin and Dziedzic^4^, Abraham radiation pressure results in an inward movement of the surface whereas Minkowski pressure results in an outward movement. Loudon’s theory on radiation pressure^5^ supports Abraham light momentum where the dielectric surface feel inward radiation pressure when light enters (or leaves) the dielectric surface which gain (or lose) a momentum of 2*E/c(n*−1) (*n*+1) [or 2*E/*(nc) (*n*−1) (*n*+1)] from light. On the contrary, Minkowski theory exhibits an outward radiation pressure when light interact with surface and the amount of momentum change of the surface will be 2*E/c(n*−1) (*n*+1) [or 2*nE/c(n*−1) (*n*+1)]. Theoretical and experimental studies over the past few years support both the theories one over the other, which kept the controversy alive^6^,^7^,^8^,^9^,^10^,^11^,^12^,^13^. The first significant experiment, in support of Minkowski momentum, has been conducted by Ashkin and Dziedzic^4^ by passing a laser beam through a glass cell containing air and water, where the outward direction of net force at the surface validate the Minkowski momentum. Attempts are made to resolve the controversy between Abraham and Minkowski by theoretical approach, identifying Abraham momentum as kinetic momentum and Minkowski momentum as the canonical momentum shown by Hinds *et al*.^8^. However most of such investigations have been conducted in air-liquid interfaces only^12^,^14^.

Is the radiation pressure of light capable of modifying the surface of a solid as well? This question is still debatable due to the absence of any direct observation of surface modification of solid occurred due to the radiation pressure of light. It has been reported that using an all-optical pump probe photo thermal method or photo thermal mirror, the radiation pressure can be detected in a transparent dielectric solid^7^. In another report, She *et al*.^15^ validate the Abraham’s momentum, where by using a silica filament (SF) it has been shown that light exerts an inward push force on the free end of silica nano-filament. However, it is also important to understand the nature of surface modification *i.e.* shape, size and its dependence on the radiation power.

In case of bulk material, the absence of suitable targets to show surface deformation due to very small change in momentum of the incident light and the complicacy to conduct such experiments are the major limitations to produce direct proof of the existence of radiation pressure^1^,^11^. However nanomaterials, especially 2D nanoflakes could be the potential candidates to demonstrate such light-matter mechanical interaction as they are intrinsically lightweight structures having very high surface to volume ratio and strong covalent structure to sustain localized radiation pressure^16^,^17^,^18^,^19^. In a report, Conti *et al*.^20^ have predicted that graphene, which is one of the strongest and lightest nanomaterial, can be a good candidate for such radiation pressure experiments and can open a new path to solve the fundamental problems like Abraham-Minkowski dilemma or Casimir effect, in which the material needs to sustain very high localized opto-mechanical pressure. Graphene derivatives have already shown potential application in ultrafast photonics such as saturable absorber for laser mode locking^21^,^22^. Mechanical properties of Graphene and GO have already been well established by using AFM, where stress is generated either by nano-indentation or by bending the flakes using AFM tips^18^,^23^. Although these experiments are successful to characterize the linear stress-strain curve for graphene films but there is no report available investigating opto-mechanical pressure on graphene or its derivatives.

Herein, we report for the first time direct observation of surface deformation on thin film of two dimensional GO due to the radiation pressure of low power laser irradiation. We have systematically examined the geometry of the deformed surface of GO films and have established its dependence on laser power. AFM analysis provides the direct evidence of inward bending of the surface due to the radiation pressure, where Raman spectra of the GO films reveal the information about the strain generated inside the film due to laser irradiation.

To investigate the effect of light radiation pressure we have irradiated GO thin film using a focused 532 nm continuous wave laser through confocal Raman Microscope arrangement and simultaneously recorded the real time Raman spectra with different laser power. The laser power has been varied using the controller micro-meter screw of the laser system which has a non-linear response (calibration curve is provided in supplementary S1). Atomic Force Microscopy (AFM) is used to investigate the change in surface morphology of the irradiated spot on GO film, where laser power dependent bending of the irradiated surface has been observed.

## Results and Discussion

**Figure 1:**
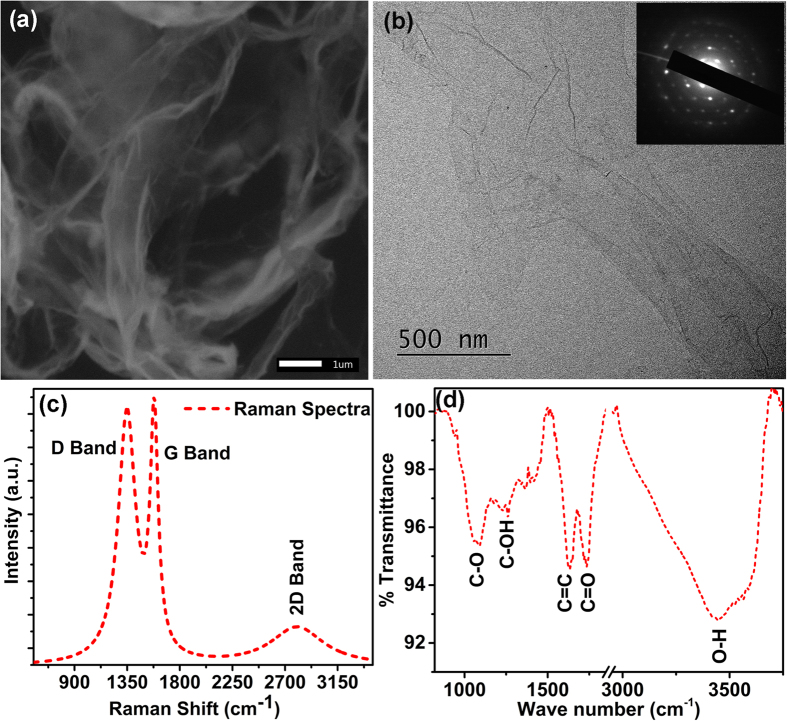
Characterization of Graphene Oxide (GO) sheets. (**a**) SEM image shows the exfoliated GO sheet. (**b**) TEM image of GO sheet; Inset in Fig. 1b shows the SAED pattern of GO. (**c**) Raman spectra of GO showing D, G and 2D band. (**d**) FTIR spectra of GO showing the presence of different functional groups.

SEM and TEM micrographs (Fig. 1a and b respectively) of the as grown GO samples exhibits few layered 2D configuration with typical wrinkled structure of the GO flakes. The electron diffraction pattern, shown in the inset of Fig. 1b, confirms the hexagonal structure of planar GO flakes. The quality of the GO sheets is characterized by Raman and FTIR spectroscopy. Three characteristics Raman peaks at ~1355 cm^−1^ (D-band), ~1580 cm^−1^ (G-band) and ~2700 cm^−1^ (2D-band) are present in the Raman spectra of the as grown GO flake (Fig. 1c). FTIR spectra (Fig. 1d) confirm the presence of different functional groups at 3430 cm^−1^ (O-H stretching vibration), 1720–1740 cm^−1^ (C=O stretching vibration), 1226 cm^−1^ (C-OH stretching vibration) and 1103 cm^−1^ (C-O stretching vibration).

**Figure 2:**
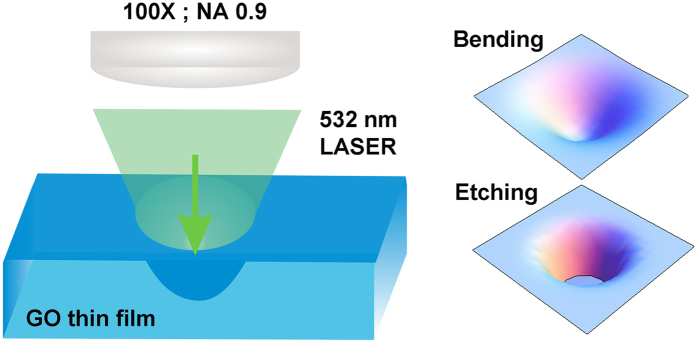
Schematic diagram of the experiment and the modification of GO solid surface. GO thin film was irradiated by a 532 nm laser beam through a 100X objective (NA 0.9). For low laser power the irradiated spot shows bending of the GO surface, whereas for high power laser irradiation etching of GO surface takes place. The GO thin film bends due to radiation pressure whereas the void formation takes place for high power laser irradiation.

Water dispersed GO flakes are drop casted on Si wafer to deposit the GO thin film on which the laser irradiation is carried out for various laser power and exposure time to conduct radiation pressure measurements. The schematic of the experimental procedure of laser irradiation and the surface modification of GO film due to radiation pressure is shown in Fig. 2. GO film is irradiated with 532 nm laser through a 100X objective. We have observed two distinct type of surface modification of the GO solid surface. At low power region (<1.59 mW), bending of the GO surface is observed whereas at higher laser power (≥1.59 mW) irradiation, etching of the GO surface is prominent, which we have explained in detail in the later part of the manuscript.

**Figure 3:**
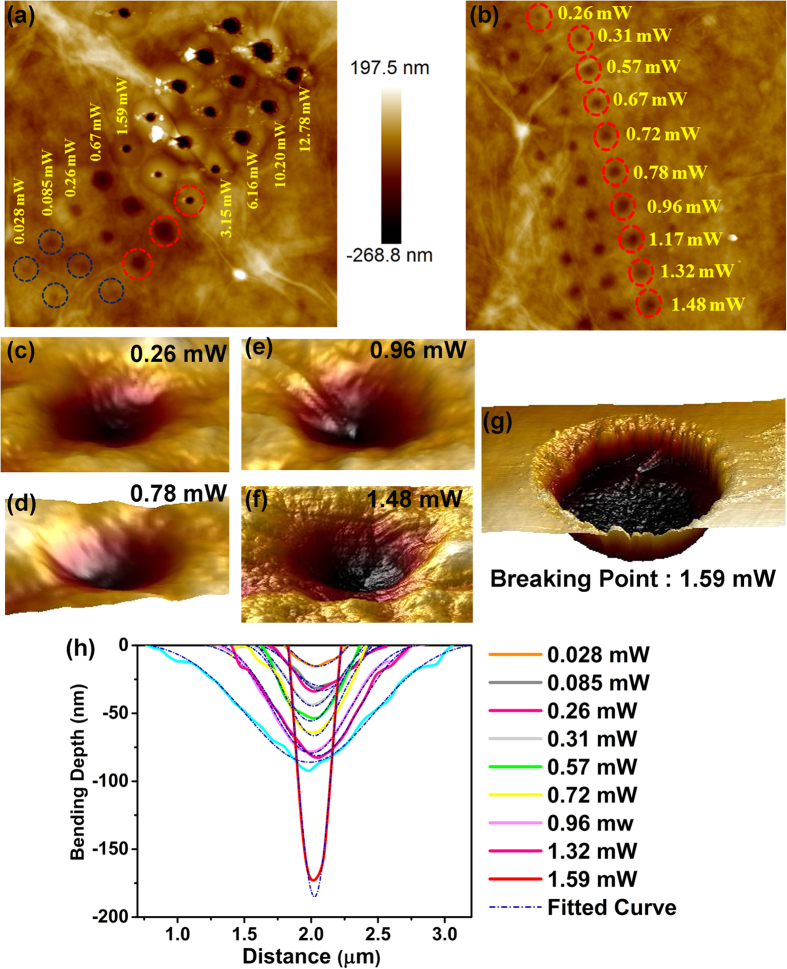
AFM investigation on the effect of laser irradiation on GO surface with 532 nm laser of different power. (**a,b**) AFM height image of GO surface after focused laser irradiation at different spots for various laser power. Bending due to 0.026 mW and 0.085 mW laser are highlighted with dark blue circles in Fig. 3a. Scale for both (**a**) and (**b**) are same. (**c–f**) High magnification 3D view of the AFM images showing bending of surface due to the laser power of 0.26 mW, 0.78 mW, 0.96 mW and 1.48 mW. (**g**) AFM image in 3D representation of the void formed while the laser power reaches breaking point at 1.59 mW, where the etching gets ignited. (**h**) Variation of bending depth and bending diameter as a function of laser power.

Figure 3(a,b) demonstrate the AFM images of surface deformation on the GO film occurred due to laser radiation pressure. Apart from AFM, the surface deformation of the GO can also be identified through optical microscope and scanning electron microscope (SEM) as demonstrated in Supplementary S2. Initially at very low laser power (<0.028 mW), the surface deformation of the GO film is negligible and the bending depth is not significant enough to distinguish it from the intrinsic surface roughness of the film. Figure 3a shows the AFM image of the GO film having the surface deformation at the laser irradiation spot for laser power varying from 0.028 mW to 12.78 mW. For each laser power, three laser shots are taken to get the average depth and diameter of the deformed area of the GO surface. To have a closer look on radiation induced bending on GO surface, measurements are further done on more number of laser irradiated spots for laser power varying from 0.26 mW to 1.48 mW (shown in Fig. 3b) where the depth varies from 34 nm to 98 nm. The AFM image shown in Fig. 3b gives in-depth information about the bending depth and diameter of the laser irradiated spots. Fig. 3(c–f) show the 3D view of the AFM image of the deformed GO surface with laser irradiation power of 0.26 mW (Fig. 3c), 0.78 mW (Fig. 3d), 0.96 mW (Fig. 3e) and 1.48 mW (Fig. 3f) with bending depth of 34 nm, 69 nm, 77 nm and 98 nm respectively. Interestingly, it has been noticed that once the laser reaches to the threshold power ~1.59 mW, the etching is initiated on GO surface due to laser irradiation. Thus in this particular case, for laser power below ~1.59 mW, the film undergoes bending on its surface due to laser radiation pressure, whereas for laser power above ~1.59 mW, the etching of GO film is initiated, which we have identified as the ‘Breaking point’. Figure 3g shows the 3D representation of the surface morphology of the void on GO film, created due to etching of the surface by 1.59 mW focused laser beam. Figure 3h shows the plots of depth profile at laser irradiated spot on GO surface for various laser power. It is confirmed from the curve fitting that the depth profile of the modified surface, below the threshold power, follows the Gaussian distribution, which is consistent with the Gaussian nature of the laser beam. However the depth profile of the etched surface due to 1.59 mW laser power follows the Lorentzian distribution. The bending of the GO surface at 0.028 mW laser power gives rise to bending depth of ~14 nm and with further increase in laser power to ~1.48 mW, the bending depth increases to ~98 nm. However when the laser power reaches the threshold power to ignite the etching of GO surface, which is the breaking point, the depth of the irradiated spot abruptly increases. It is observed that for 1.59 mW laser irradiation, the depth jumps to 165 nm, creating a void (Fig. 3g) on the GO surface. Throughout all these experimental results it is very clear that the laser radiation results bending of the GO surface and the bending is in the *inward* direction of the solid surface. The radiation pressure experienced due to photon momentum transfer to the GO film, bends the surface in the direction of propagation of light, which supports the theory of Abraham over Minkowski.

**Figure 4:**
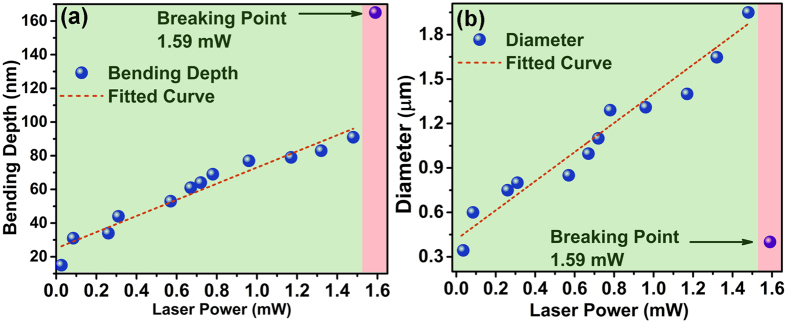
Plots of bending depth and diameter vs. laser power. Graph shows the changes in (**a**) depth and (**b**) diameter of the laser irradiated spots as a function of laser power. Dotted line indicates the fitted plot of depth and diameter with variable laser power. Both depth and diameter shows linear dependence with laser power whereas this linearity breaks at 1.59 mW laser power, which is the breaking point.

**Figure 5:**
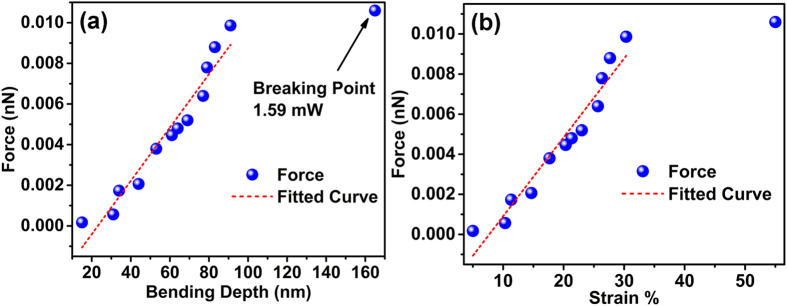
Stress-strain curve of the GO system. (**a**) Force vs. bending depth and (**b**) Force vs. strain curve shows a linear dependence below the breaking point; calculated spring constant is ~1.31 X 10^−4^ N/m.

The theoretical formulation on the effect of laser power with the bending depth is well established by several research groups^12^,^14^ for both inward and outward movement of the surface as a consequence of the Abraham or Minkowski theory of light momentum. The depth of the deformed surface and the change in diameter varies linearly with the change in laser power for both the inward and outward movement of the surface, which is an established theory for air-water interface^6^,^12^. In support of Minkowski momentum, Verma *et al*.^12^ have shown that the relation between bulge height and laser power as 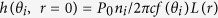, where h is bulge height at the centre, *P*_0_ is the power of laser used, *n*_*i*_ is refractive index of air-water interface, *θ*_*i*_ is the angle of incidence. Whereas Zhang *et al*.^6^ have shown that the height at the centre of the spot relates to the laser power as 
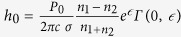
. Here the gamma function is a positive factor thus the height of the surface will be negative *i.e.* the surface will bend towards inward direction as a consequence of Abraham pressure. However, all these experimental calculations^4^,^6^,^11^,^12^ have used air-water interfaces, which is different from our study on interface of air and solid surface of GO films. In case of air-water interface the refractive index plays an important role in controlling the surface height since the major part of the light transmits through the 2^nd^ medium. However in our study we can avoid the effect of transmission as reflective property of GO film on Si substrate is dominant rather than the transmission. So neglecting the transmission effect, the expression of *h*_0_ becomes simpler and we can consider the refractive index part as a constant. This will lead to vary *h*_0_ linearly with the applied laser power. We have thoroughly measured the bending depth of each laser irradiated spot as well as the diameter of the bending area by using AFM. Figure 4(a,b) show the plots of depth and diameter of the bending dip respectively with the variation of laser power. It leads us to the conclusion that the bending depth and the diameter of the laser irradiated spot initially varies linearly with the radiation power, until the laser power reaches the breaking point where the etching of GO surface is ignited, leading to abrupt increase in the depth and decrease in the bending diameter. The linear dependence of these two parameters (depth and diameter) fits well with previous experimental results reported by other groups to probe radiation pressure of light^6^,^11^,^12^. It has already been reported and well accepted in scientific community that in case of air-water interface the relation between strain (bending depth) and the change in stress (variation in laser power) should have linear dependence^6^,^12^. In our case, the laser radiation pressure acts as stress on the GO film and creates strain by bending the surface towards inward direction of the GO surface. As stress is enhanced with increasing laser power the bending diameter and depth increases. From Fig. 4(a,b) it is quite evident that the relation between stress and strain is linear for laser power lesser than the breaking point (at 1.59 mW laser power). At 1.59 mW laser power, the diameter and depth of the GO films changes abruptly, where the bending diameter changes from 1.3 μm to 0.4 μm, while the bending depth increases from 98 nm to 165 nm. At the breaking point, the diameter of the bending dip decreases abruptly due to release of the strain on the crystal structure while the etching is initiated. We have also calculated the radiation force applied on the GO surface, obtained simply by using the formula F = 2 P⁄c, where P is the laser power and c is the velocity of light. Figure 5a shows the force-distance curve for the above mentioned system, whereas Fig. 5b shows the stress-strain curve of the system. The applied force (or stress) and the bending depth (or strain) exhibit excellent linearity below the breaking point where the applied force is 0.01 nN. At the breaking point (1.59 mW laser power or 0.01 nN force), the depth increases abruptly to 165 nm. The spring constant calculated from the force-distance curve (Fig. 5a) is ~1.31 × 10^−4^ N/m for the GO thin film. Figure 5b shows the stress-strain (or force-strain) curve of the laser irradiated GO system which shows the linear behaviour of the stress-strain curve below the breaking point.

Another significant aspect of these experiments is that for the first time we have demonstrated a novel method to create nano-patterns on GO surface by direct laser writing using very low power (minimum 1.59 mW) continuous focused laser beam. By focusing the laser with 100X objective on GO surface we have successfully achieved the minimum spot size of ~300 nm, which is very close to the diffraction limit of the 532 nm laser according to Abbe diffraction limit for a microscope [λ/(2x(NA))]. Previous attempts by other research groups have demonstrated etching of GO by using high power femto-second laser with focused laser beam of lower wavelength which limits its resolution to sub-micron range^24^,^25^.

**Figure 6:**
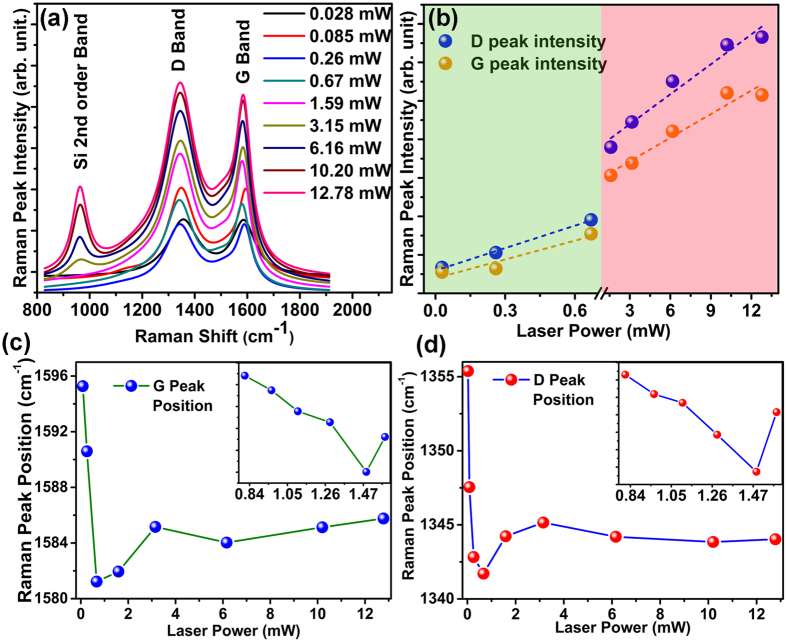
Evolution of Raman spectra due to laser radiation pressure. (**a**) Raman spectra at different laser power ranging from 0.028 mW to 12.78 mW. (**b**) Plot of D & G peak intensity with the variation of laser power. The difference in rate of change in peak intensity distinguishes bending and etching phenomena below and above the breaking point respectively (**c,d**) Change in Raman peak position (both D and G band) with respect to laser power. Both D and G peak shows similar trends of change in position with the laser power. Inset shows the zoomed view of the breaking point showing the change in Raman peak position with the laser power ranging 0.67–1.59 mW.

As mentioned in the previous section, simultaneous Raman spectroscopy has been carried out while irradiating the GO film with focused laser of various laser power. A grating of 1800 grooves/mm was used to investigate the spectral distribution of the scattered light coming from GO film through the confocal arrangement of the microscope. Figure 6a shows the Raman spectra of the GO films for different laser power in which the prominent peaks are D (~1350 cm^−1^) and G (~1585 cm^−1^) band. Also second order peak of Si is prominent for laser irradiation power higher than breaking point (>1.59 mW). The D peak corresponds to the vibration mode due to crystallographic defects on graphene carbon ring (A^1g^) whereas the G peak is due to the C-C vibration mode (E^2g^)^26^. In recent past, some efforts have been made to investigate mechanical properties of graphene by using Raman spectroscopy, where mechanical strain has been created on graphene by bending of polymer substrate or by nano-indentation method and the effect of uniaxial strain on Raman Spectra has been investigated^23^,^27^,^28^,^29^,^30^. Gómez-Navarro *et al*.^29^ have investigated the elastic properties of chemically derived graphene sheets, where the applied force and the AFM tip induced bending depth obeys the linear relation for incremental deformation. Using Raman spectroscopy we have intended to investigate the mechanical strain on GO film, originated from the laser radiation pressure. Raman spectrum of GO film for laser irradiation power varying from 0.028 mW to 12.78 mW confirms significant change in peak intensity and the peak position for both D-band & G-band as shown in Fig. 6a. The plots of relative peak intensity for D-band and G-band for various laser irradiation power are shown in Fig. 6b, which shows clear linear dependency. From the AFM results, as demonstrated in previous section, we have already confirmed that the bending due to laser radiation pressure on GO film is visible significantly for laser power range ≥0.028 mW and <1.59 mW, whereas the etching process starts after laser power reaches 1.59 mW. Comparable conclusion reflects in Fig. 6b where the rate of change in D peak intensity [I(D)] for the laser power ranging from 0.028 mW to 0.67 mW is 1.94 arb. unit/mW whereas beyond that range the value drops to 0.25 arb. unit/mW. Similar kind of response is observed for the G peak intensity [I(G)] as well, where the rate of change of I(G) value jumps from 1.62 arb. unit/mW to 0.20 arb. unit/mW. Nearly 8-fold decrease in rate of change of intensity can be well understood if we divide the total system into two distinguishable parts. The first part is marked with green colour in Fig. 6b which shows faster rate of change in intensity and occurs while the laser radiation bends the surface. The second part, marked with red colour in Fig. 6b with slower rate of change in intensity of I(D) and I(G) occurs when the laser power reaches the breaking point and the etching of the GO surface is initiated. Thus the Raman spectroscopy can be used to find the breaking point where the bending due to laser irradiation stops and the etching of GO starts. In addition it can also be noticed from the Fig. 6a that the second order Si peak at ~900 cm^−1^ becomes prominent after the laser power crosses 1.59 mW, as the Si substrate get exposed to the laser radiation due to the etching of GO film.

More detailed information about the laser radiation pressure induced strain on the GO film, can be explored by a closer view of the peak position of the Raman spectra. Since G-band plays an important role to characterize carbon nanomaterials, its peak position has been investigated by various groups to understand its dependence on applied uniaxial strain^27^,^28^,^31^. Although there are few reports available regarding the effect of applied strain on Raman spectra of graphene, Mohiuddin *et al*.^27^ have described well regarding the evolution of G peak position with the applied uniaxial strain. The shear component of the strain splits the G band into G^+^ and G^−^ peaks when the graphene surface bends and the hydrostatic component of the strain shifts the G peak at a rate of 10.8 cm^−1^/% for G^+^ or 31.7 cm^−1^/% for G^−^ mode. In another report^28^, the rate of change of G^+^ and G^−^ peak position with the applied strain is found to be 12.5 ± 2.6 cm^−1^/% and 5.6 ± 1.2 cm^−1^/% respectively. Although both the experiments have followed similar kind of technique to apply uniaxial strain but there is a significant difference in the rate of change of G peak (for both G^+^ and G^−^) position. However, the trend followed by the G peak is similar to both the cases upon applied strain. In our experiments the light is incident on the sample in normal direction, so we can ignore the infinitesimal shear component of the strain produced due to radiation pressure which fits well with the Raman results (Fig. 6) of the GO film as no significant splitting of the G mode is found. Figure 6c demonstrates that initially the G peak position shows red shifts due to the strain generated by the radiation pressure of light as a result of elongated bond length originated due to the bending of the GO surface. However when the laser power reaches the breaking point (1.59 mW) where the etching starts, the uniaxial strain is released due to relaxation of the crystal structure of GO and a sudden blue shift occurs in the G-peak position. This response matches very well with the previous reported results^16^,^29^, where red shift occurred due to uniaxial strain on graphene and the response flipped beyond the breaking point. We have noticed similar kind of response with the D-peak position as well, shown in Fig. 6d which implies the whole spectra shifts while experiencing radiation pressure of laser light. Thus Raman spectroscopy could be an important tool to qualify and quantify the presence of radiation pressure of light on solid surface.

## Conclusion

In summary, we have successfully demonstrated an inward bending of GO thin film surface, due to low laser power irradiation of a 532 nm focused laser beam, which follows Abraham theory of light radiation pressure. Bending of GO thin film has been noticed for laser power below 1.59 mW and beyond which the laser beam ignites etching of the GO surface. The above mentioned inward bending and etching of GO surface has been thoroughly investigated by using AFM and Raman spectroscopy. AFM studies reveal that for very low laser power, ranges from 0.028 mW to 0.67 mW, the surface of the irradiated spot undergoes bending due to light radiation pressure with bending depth varying linearly from 14 nm to 97 nm. Abrupt change in bending depth and diameter at 1.59 mW laser power breaks the linearity which has been identified as ‘Breaking point’. Similar kind of distinguishable response occurs in the rate of change of Raman intensity for both the G-band and D-band where 8-fold decrease in the intensity is observed after the incident laser power reaches the breaking point. The rate of change in Raman intensity confirms that focused laser beam modifies the surface in two different ways; initially bending of the surface due to radiation pressure and finally etching of the surface due to laser irradiation. With the variation of laser power, the peak position of G-band and D-band initially shows red shift due to uniaxial strain induced in the GO thin film, however a sudden blue shift can be observed as the laser power reaches the breaking point where the uniaxial strain is released abruptly due to etching of GO layers. Our results suggest that the effect of radiation pressure on GO thin film follow Abraham model of radiation pressure or momenta of light, which is the first experimental observation of radiation pressure on any solid nanomaterial surface. These experimental results enlighten the age-old controversy Abraham-Minkowski dilemma and may also pave a path towards the novel route of nanopatterning of graphene derivatives for various electronic and optical applications.

## Methods

### Materials

Graphite powder was purchased from Alpha Aesar. Potassium permanganate (KMnO_4_), sulphuric acid (H_2_SO_4_), hydrogen peroxide (H_2_O_2_), hydrochloric acid (HCl), ethanol were received from Merck. Phosphoric acid (H_3_PO_4_) was purchased from TCI chemicals. Throughout the experiments double distilled de-ionized water (DI water) was used. All reagents were of analytical grade and used without further purification.

### Preparation of Graphene Oxide (GO)

The graphene oxide flakes are prepared by modified Hummer’s method^32^. Briefly, 9:1 mixture of concentrated H_2_SO_4_ (120 mL) and H_3_PO_4_ (13 mL) are added to a mixture of Graphite (1 g) and KMnO_4_ (6 g) powder. The solution is heated to 55 °C and kept in stirring condition for 12 hrs. Then the solution is cooled to room temperature and poured into 400 mL ice cooled DI water with 30% H_2_O_2_. Exfoliated GO precipitation is collected after centrifugation followed by washing in DI water, 30% HCl and ethanol for three times each. As synthesized GO flakes in powder form are dried in 80 °C for 12 hrs and then dispersed in DI water. The dispersed GO flakes are simply drop casted on the Si substrate and dried in an oven at 150 °C for 2 hrs.

### Measurements

The thickness of the thin film is monitored by profilometer (Bruker DektakXT) which is ~300 nm. Both the AFM and Raman studies are conducted on the same GO thin film, homogeneously distributed on the Si substrate. A Raman spectrometer system (Witec alpha 300 R) is used to irradiate the GO film with focused laser coming from 100X objective of a confocal microscope. An Nd:YAG continuous laser of 532 nm wavelength is used to irradiate the GO surface with varying laser power. Real time Raman signals are recorded which is discussed in detail in the manuscript. Surface deformation of the GO films is investigated by Bruker Multimode AFM system in ‘tapping mode’ which reveals in-depth information about the shape and size of the deformed surface of GO films, showing the dependence on the irradiated laser power. Laser irradiations on the GO films as well as all characterizations are conducted in an ambient environment. Laser power is calibrated with the micro-meter scale, present in the laser source, using Thorlabs optical power meter (PM100D). The calibrated laser power with the micro screw is shown in Fig. S1. The morphology of the GO sample was characterized by JEOL JSM-IT300 scanning electron microscope and JEOL JEM2100 transmission electron microscope. FTIR spectroscope (Agilent Cary 660 FTIR) was used to characterize the functionalization of the GO surface.

## Additional Information

**How to cite this article**: Kundu, A. *et al*. Graphene Oxide Demonstrates Experimental Confirmation of Abraham Pressure on Solid Surface. *Sci. Rep.* **7**, 42538; doi: 10.1038/srep42538 (2017).

**Publisher's note:** Springer Nature remains neutral with regard to jurisdictional claims in published maps and institutional affiliations.

## Supplementary Material

Supplementary Information

## References

[b1] García-Segundo C., Ramos-Ortiz G. & Villagrán-Muniz M. Experimental evidence for radiation pressure on a macroscopic dielectric. Optics Communications 225, 115–122 (2003).

[b2] Minkowski H. Die Grundgleichungen für die elektromagnetischen Vorgänge in bewegten. Nachr. Königl. Ges. Wiss. Göettingen 53–111 (1908).

[b3] Abraham M. Zur Elektrodynamik bewegter Krper. Rend. Circ. Mat. Palermo. 28, 1–28 (1909).

[b4] Ashkin A. & Dziedzic J. M. Radiation Pressure on a Free Liquid Surface. PhysRevLett. 30, 139–142, (1973).

[b5] Loudon R. Theory of the radiation pressure on dielectric surfaces. Journal of Modern Optics 49, 821–838 (2002).

[b6] Zhang L., She W., Peng N. & Leonhardt U. Experimental evidence for Abraham pressure of light. New Journal of Physics 17, 053035 (2015).

[b7] Astrath N. G. C., Lukasievicz G. V. B., Malacarne L. C. & Bialkowski S. E. Surface deformation effects induced by radiation pressure and electrostriction forces in dielectric solids. Applied Physics Letters 102, 231903 (2013).

[b8] Hinds E. A. & Barnett S. M. Momentum Exchange between Light and a Single Atom: Abraham or Minkowski? Physical review letters 102, 050403 (2009). 19257490 10.1103/PhysRevLett.102.050403

[b9] Pfeifer R. N. C., Nieminen T. A., Heckenberg N. R. & Rubinsztein-Dunlop H. Colloquium: Momentum of an electromagnetic wave in dielectric media. Reviews of Modern Physics 79, 1197–1216 (2007).

[b10] Loudon R., Barnett S. M. & Baxter C. Radiation pressure and momentum transfer in dielectrics: The photon drag effect. Physical Review A 71, 063802 (2005).

[b11] Astrath N. G., Malacarne L. C., Baesso M. L., Lukasievicz G. V. & Bialkowski S. E. Unravelling the effects of radiation forces in water. Nature communications 5, 4363 (2014). 10.1038/ncomms5363 PMC4102109 24999561

[b12] Verma G. & Singh K. P. Universal long-range Nanometric Bending of Water by Photons Momentum. Physical review letters 115, 143902 (2015). 26551814 10.1103/PhysRevLett.115.143902

[b13] Barnett S. M. Resolution of the abraham-minkowski dilemma. Physical review letters 104, 070401 (2010). 20366861 10.1103/PhysRevLett.104.070401

[b14] Walker G. B. & Lahoz D. G. Experimental observation of Abraham force in a dielectric. Nature 253, 339 (1975).

[b15] She W., Yu J. & Feng R. Observation of a push force on the end face of a nanometer silica filament exerted by outgoing light. Physical review letters 101, 243601 (2008). 19113619 10.1103/PhysRevLett.101.243601

[b16] Zandiatashbar A. . Effect of defects on the intrinsic strength and stiffness of graphene. Nature communications 5, 3186 (2014). 10.1038/ncomms4186 24458268

[b17] Lee C., Wei X., Kysar J. W. & Hone J. Measurement of the elastic properties and intrinsic strength of monolayer graphene. Science 321, 385–388 (2008). 18635798 10.1126/science.1157996

[b18] Poot M. & van der Zant H. S. J. Nanomechanical properties of few-layer graphene membranes. Applied Physics Letters 92, 06311 (2008).

[b19] Lee G. H. . High-strength chemical-vapor-deposited graphene and grain boundaries. Science 340, 1073–1076 (2013). 23723231 10.1126/science.1235126

[b20] Conti C. & Boyd R. Nonlinear optomechanical pressure. PhysRevA. 89, 033834 (2014).

[b21] Zhang Z. . Microwave and optical saturable absorption in graphene. Optics Express 20, 23201 (2012). 23188285 10.1364/OE.20.023201

[b22] Zhang H., Bao Q., Tang D., Zhao L. & Loh K. Large energy soliton erbium-doped fiber laser with a graphene-polymer composite mode locker. Applied Physics Letters 95, 141103, (2009).

[b23] Suk J. W. Piner R. D. An J. & Ruoff R. S. Mechanical Properties of Monolayer Graphene Oxide. ACS Nano 4, 6557–6564 (2010). 20942443 10.1021/nn101781v

[b24] Zhang Y. . Direct imprinting of microcircuits on graphene oxides film by femtosecond laser reduction. Nano Today 5, 15–20 (2010).

[b25] Zhang Y.-L., Chen Q.-D., Xia H. & Sun H.-B. Designable 3D nanofabrication by femtosecond laser direct writing. Nano Today 5, 435–448 (2010).

[b26] Ferrari A. C. & Basko D. M. Raman spectroscopy as a versatile tool for studying the properties of graphene. Nature nanotechnology 8, 235–246 (2013). 10.1038/nnano.2013.46 23552117

[b27] Mohiuddin T. M. G. . Uniaxial strain in graphene by Raman spectroscopy:Gpeak splitting, Grüneisen parameters, and sample orientation. Physical Review B 79 (2009).

[b28] Huang M. . Phonon softening and crystallographic orientation of strained graphene studied by Raman spectroscopy. Proceedings of the National Academy of Sciences of the United States of America 106, 7304–7308 (2009). 19380746 10.1073/pnas.0811754106 PMC2678610

[b29] Gómez-Navarro C. Burghard M. & Kern K. Elastic Properties of Chemically Derived Single Graphene Sheets. Nano Letters 8, 2045–2049 (2008). 18540659 10.1021/nl801384y

[b30] Yu T. . Raman Mapping Investigation of Graphene on Transparent Flexible Substrate: The Strain Effect. The Journal of Physical Chemistry C 112, 12602–12605 (2008).

[b31] Liu L., Zhang J., Zhao J. & Liu F. Mechanical properties of graphene oxides. Nanoscale 4, 5910–5916 (2012). 22898942 10.1039/c2nr31164j

[b32] Marcano D. C. . Improved Synthesis of Graphene Oxide. ACS Nano 4, 406–4814 (2010). 10.1021/nn1006368 20731455

