# Supplementary material for: Graphene Oxide Demonstrates Experimental Confirmation of Abraham Pressure on Solid Surface
Source: Sci Rep. 2017 Feb 13;7:42538. doi: 10.1038/srep42538 (PMC5304167; doi:10.1038/srep42538)
Supplement: Supplementary Information [file srep42538-s1.pdf]

Graphene Oxide Demonstrates Experimental Confirmation of Abraham Pressure on Solid Surface

Anirban Kundu, Renu Rani and Kiran S Hazra*
Institute of Nano Science and Technology
Habitat Centre, Phase 10, Sector 64, Mohali, Punjab-160062, India
*kiran@inst.ac.in

Supplementary Figure S1:

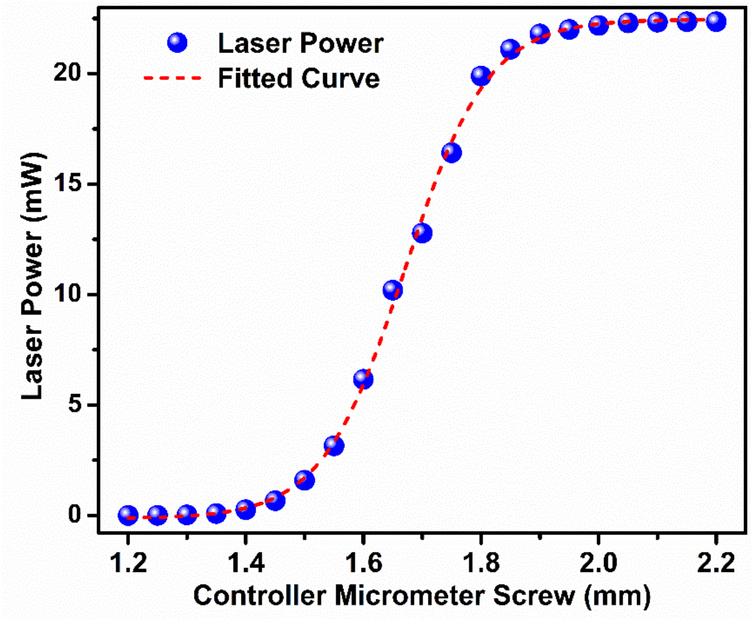

Figure S1| Calibration curve of laser power with controller micrometer screw. It shows the non-linearity response.

Supplementary Figure S2:

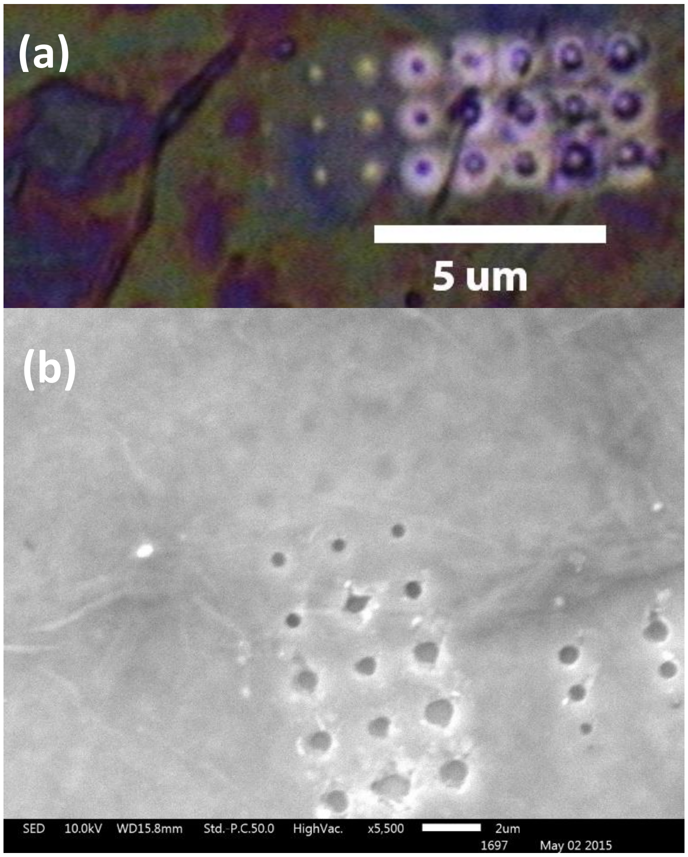

Figure S2| (a) Optical and (b) SEM images of the deformed Graphene Oxide (GO) surface due to irradiation of different laser power. The laser power varies from 0.026 mW to 12.78 mW.
